# The relationships among *Leishmania infantum* and phyllostomid bats assessed by histopathological and molecular assays

**DOI:** 10.1016/j.ijppaw.2023.100904

**Published:** 2023-12-30

**Authors:** Alanderson Rodrigues da Silva, Heitor Miraglia Herrera, Carina Elisei de Oliveira, Jaire Marinho Torres, Ana Maria Reis Ferreira, Juliana da Silva Leite, Rodrigo Caldas Menezes, Érica Verneque Martinez, Gabrielly Moreira dos Santos de Oliveira, Filipe Martins Santos, Gisele Braziliano de Andrade

**Affiliations:** a Universidade Católica Dom Bosco, Programa de Pós-graduação em Biotecnologia, Av. Tamandaré, 6000, CEP: 79117900, Campo Grande, Mato Grosso do Sul, Brazil; b Universidade Católica Dom Bosco, Programa de Pós-graduação em Ciências Ambientais e Sustentabilidade Agropecuária, Av. Tamandaré, 6000, CEP: 79117900, Campo Grande, Mato Grosso do Sul, Brazil; c LAMP LAB - LAMP Diagnostico LTDA– Av. Tamandaré, 6000, Jardim Seminário, 79117-900, Campo Grande, Mato Grosso do Sul, Brazil; d Universidade Federal Fluminense, Departamento de Patologia e Clínica Veterinária, Faculdade de Veterinária, Rua Vital Brazil, 64, Niterói, Rio de Janeiro, Brazil; e Fundação Oswaldo Cruz, Laboratório de Pesquisa Clínica em Dermatozoonoses em Animais Domésticos, Av. Brasil, 4036, Sala 119, Manguinhos, Brazil; f Instituto Nacional de Infectologia Evandro Chagas, Rio de Janeiro, Rio de Janeiro, Brazil; g Universidade Católica Dom Bosco, Programa Institucional de Bolsas de Iniciação Científica, Av. Tamandaré, 6000, CEP: 79117900, Campo Grande, Mato Grosso do Sul, Brazil

**Keywords:** Chiropteran, Histopathology, Immunohistochemistry, Dead-end host, Leishmaniasis

## Abstract

Bats have been reported as reservoir host of *Leishmania* spp. worldwide, mostly by molecular detection. However, it is still unclear whether bats act as reservoirs of *Leishmania infantum* to sandflies vectors. In this sense, the investigation of amastigotes forms in the target organs, and the characterization of their associated inflammation, may help to clarify the epidemiological importance of bats in endemic areas for leishmaniasis. The aim of this work was to investigate the host-parasite relationships under microscopic evaluation and predict the epidemiological role of two phyllostomid bats species naturally infected by *L. infantum* in an endemic area for human leishmaniasis. Fragments of skin, liver and spleen of *L. infantum* positive and negative bats (*Artibeus planirostris* and *Carollia perspicillata)* by qPCR, were studied by histological and immunohistochemical techniques. Both groups, positive and negative, did not show differences in the histopathological study, presenting only discrete tissue changes. Liver and skin showed mild inflammatory reactions. Findings on spleen consisted of reactivity of the lymphoid follicles, expressive presence of apoptotic cells and macrophages containing abundant phagocytic cells debris. We did not find amastigote forms in tissues by histological and IHC techniques in positive qPCR bats. Our results allow us to hypothesize that phyllostomid bats seem to have an important role in reducing the risk of transmission, possibly acting as dead-end host.

## Highlights

• Relationship between phyllostomid bats and *Leishmania infantum* at microenvironment.
• Amastigote forms were not detected in the skin, liver and spleen tissues of phyllostomid bats positive for *L. infantum* by PCR.
• Phyllostomid bats may act as dead-end hosts for *L. infantum*.

## Graphical abstract

**Figure:**
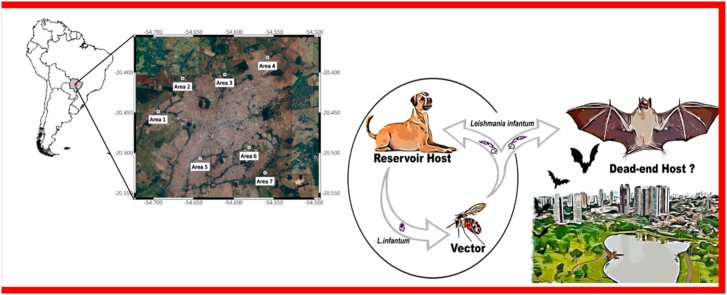

## 1 Introduction

Several authors have reported that bats play an important role in the epidemiology of leishmaniasis in Americas since *L.* *infantum* DNA have been detected in these flying mammals (Savani et al., 2010; Shapiro et al., 2013; Berzunza-Cruz et al., 2015; de Rezende et al., 2017; Castro et al., 2020; de Araújo et al., 2022). A single report of *L. infantum* isolation in Seba's short-tailed bat (*Carollia perspicillata*) (Lima et al., 2008) opens questions that must be carefully addressed about the epidemiological role of bats in the epidemiology of visceral leishmaniasis.

The study of the presence or absence of *Leishmania* spp. amastigotes forms in the target tissues, as well as the characterization of their associated inflammation may help to clarify the epidemiological importance of hosts species. Considering wildlife, the interactions between *L. infantum* and free-living mammals are poorly studied due to the complexity of monitoring naturally infected animals, as well as the difficulties of maintaining wild animals for experimental investigations. The studies approaching bats and carnivores naturally infected by *L. infantum* (Courtenay et al., 2002; Lima et al., 2008; Figueiredo et al., 2008; Cavalera et al., 2020; Vieira et al., 2022), as well as opossum and small rodent experimentally parasitized (Sherlock et al., 1988; Roque et al., 2010), have shown neither clinical symptoms nor lesions compatible with visceral leishmaniasis as described in dogs. Although positive bats for *L. infantum* may constitute a source of infection for Phlebotomine vectors, there are no studies that confirm their competence in the transmission and consequently role of these flying mammals in the leishmaniasis epidemiology.

The gold standard methods for diagnostic of *Leishmania* spp. include culture of hematopoietic and lymphoid tissues, however it demonstrates low sensitivity (Ortega et al., 2017; Sundar and Singh, 2018). The molecular techniques, such as conventional polymerase chain reaction (PCR) and real-time quantitative PCR (qPCR) have become important diagnostic tools due to their high sensitivity, specificity, and applicability in diverse biological samples (Galluzzi et al., 2018). Additionally, histopathological and immunohistochemistry (IHC) assays are tools for detection of the parasite and related antigens in areas of inflammation and tissue damage (Abrantes et al., 2016; Headley et al., 2019; Gonzalez et al., 2019). All together, these techniques contribute to the understanding of the pathogenesis of leishmaniasis. We aimed to study the histopathological changes in skin, liver, and spleen of *Artibeus* *planirostris* and *C. perspicillata* naturally infected by *L. infantum* to better understand the host-parasite relationships and the epidemiological role of the two common species of phyllostomid bats in an endemic area for visceral leishmaniasis at Brazilian Midwest.

## 2 Materials and methods

The biological samples of *A. planirostris* and *C. perspicillata* were obtained by convenience sampling from bats captured in forest remnants of Campo Grande (20°27′22.40″S 54°36′38.25″W), Mato Grosso do Sul, an endemic area to visceral leishmaniasis (Harhay et al., 2011), between June 2017 and January 2018. For capture, eight nets (12.0 × 2.5 m) were placed in seven forest fragments, including urban parks and residential villages. The nets were opened at sunset for the next 6 h, for seven to eight nights per area, totaling 53 nights of capture. The global capture effort ranged from 10,080 h.m2 to 11,520 h.m2, totaling 76,320 h.m2. On each sampling night, a maximum of two individuals of each species was removed, any other animals captured were identified and released at the capture site. The trapped bats were identified in the field according to Reis et al. (2017). The procedures for capturing animals and collecting biological samples followed specifications presented in Brazilian Biodiversity Authorization and Information System (Sisbio license n ° 56,804-1) and authorization by the Ethics Committee for the Use of Animals of Universidade Católica Dom Bosco n ° 006/2017.

Collected bats were anesthetized with 9:1 ketamine chlorydrate (10%) and acepromazine (1%) association and euthanized. Spleen fragments were collected and fixed in ethanol for detection of *L. infantum* by molecular tests. The DNA was extracted from spleen using QIAamp DNA Mini Kit Qiagen, Venlo, The Netherlands. Additionally, spleen, liver and skin samples were collected and fixed in buffered formalin for histopathological and immunohistochemical tests.

Molecular tests were performed using a PCR assay with oligonucleotides to minicircles of *Leishmania* spp. kDNA and a variable region of the trypanosome 18S rRNA gene (Roque et al., 2010; de Rezende et al., 2017). Positive samples for *Leishmania* sp. were analyzed using qPCR with *L. infantum*-specific primers, based on the study of Francino et al. (2006). All PCR reactions included sterile distilled water instead of DNA as negative control. The *L.infantum* strain LHV14 used as positive control was kindly provided by Dr. João Santana, head of the immunoparasitology laboratory at the University of São Paulo, Ribeirão Preto (USP/Ribeirão Preto).

The tissue samples of all *L. infantum* positive bats, and ten individuals of each bat species negative for *L. infantum* were processed and embedded in paraffin, cut into sections (4 μm thick) and stained by hematoxylin and eosin technique (H&E) (Luna, 1968). The histopathological analyzes were carried out using an Axio Scope. A1® microscope (Carl Zeiss Microscopy GmbH, Jena, Germany) with the aid of Zeiss Zen® Lite software for photomicrographs capture. Classification of spleen lesions was based on the degree of the structural organization of the white pulp (WP) as described by Santana et al. (2019). Immunohistochemistry (IHC) assay for *Leishmania* spp. was performed in the wing skin, liver, and spleen fragments of qPCR positive samples according to the standardization made by Boechat et al. (2016). *Leishmania infantum* positive dog skin was used as positive control and PCR *Leishmania* negative bat skin as negative control.

## 3 Results

Forty-nine *A. planirostris* (12 females and 37 males) and 50 *C. perspicillata* (18 females and 32 males) were collected. The nPCR results showed ten (3 females and 10 males) *A. planirostris* and six (2 females and 4 males) *C. perspicillata* positive for *Leishmania* spp. by nPCR that were also positive for *L. infantum* according to qPCR (Supplementary material 1). Discreet alopecia in the wing skin of both positive and negative animals was observed. Amastigotes forms were not detected in the wing skin, liver, and spleen by both H&E and IHC techniques, even in the qPCR positive animals.

**Fig. 1:**
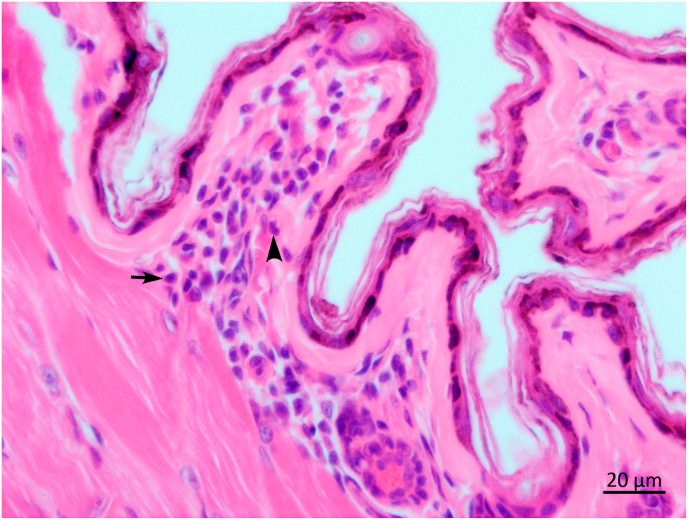
Photomicrography of wing skin of an adult male *Artibeus planirostris* qPCR *Leishmania infantum* positive. No amastigotes forms were found, only unspecific mild mixed inflammatory infiltrate of dermis with mononuclear (arrow) and polymorphonuclear cells (arrowhead), H&E, 40x objective.

**Fig. 2:**
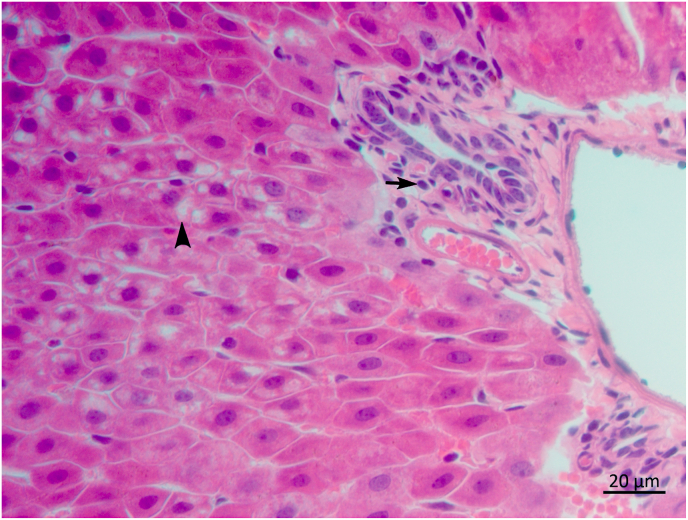
Photomicrography of liver of an adult female *Leishmania infantum* negative *Carollia perspicillata* presenting cytoplasmic vacuolation of hepatocytes (arrowhead) and mild lymphocytic infiltrate of portal area (arrow), H&E, 40x objective.

**Fig. 3:**
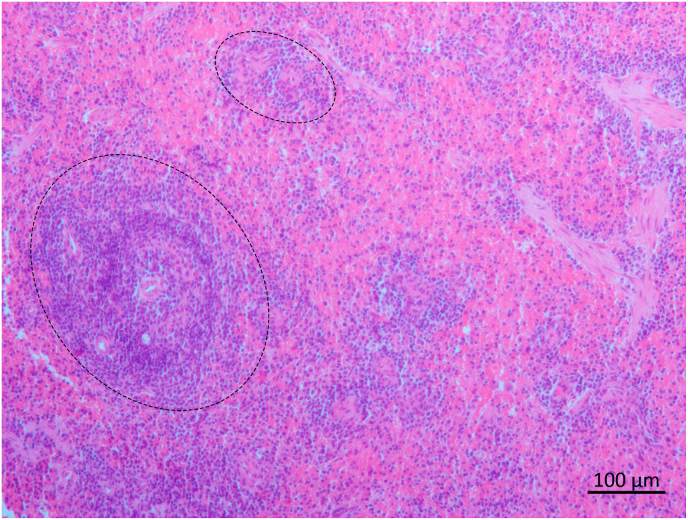
Photomicrography of spleen of an adult male *Artibeus planirostris* qPCR *Leishmania infantum* positive. No amastigotes forms were found, only unspecific mild hyperplasia (big ellipse) and hypoplasia (small ellipse) of lymphoid follicles in the reactive white pulp. Note the lack of delimitation between the WP and red pulp (RP), H&E, 10x objective. (For interpretation of the references to colour in this figure legend, the reader is referred to the Web version of this article.)

**Fig. 4:**
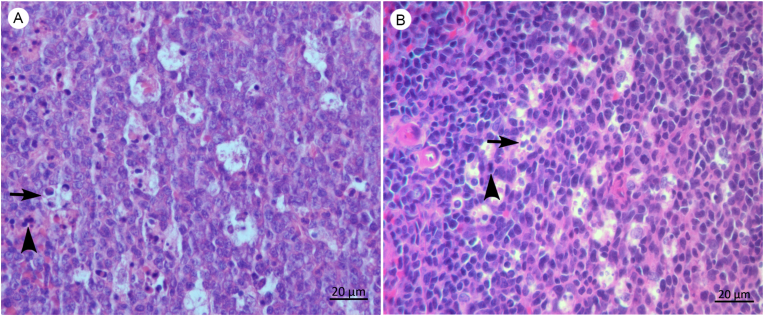
Photomicrography of spleen of an adult male *Artibeus planirostris* (A) and *Carollia perspicillata* (B) qPCR *Leishmania infantum* positive. No amastigotes forms were found. Note tingible bodies macrophages in the germinal center containing phagocytic apoptotic cells (arrow) and apoptotic cells (arrowhead), H&E, 40x objective.

Moreover, we did not observe any difference among positive and negative animals regarding histopathological analysis. Skin revealed focal inflammation in the wing membranes and dermis of *A. planirostris* and *C. perspicillata,* mainly evidenced by the presence of a mixed inflammatory infiltrate (Fig. 1). Common findings in liver analysis were cytoplasmic vacuolation of hepatocytes and minor inflammatory reaction characterized by scarce lymphocytes in the hepatic portal area (Fig. 2). In the spleen, we observed reactive WP evidenced by the presence of mild hyperplasia and hypoplasia of lymphoid follicles with a slight change of architecture and disappearance of the marginal zone, demonstrated by the lack of delimitation between the WP and red pulp (RP) (Fig. 3). Furthermore, we observed several apoptotic cells in the marginal zone of the WP and autophagy, expressed by abundant tingible body macrophages (TBMs) containing phagocytic cells debris in the germinal center (Fig. 4).

## 4 Discussion

Bats have been reported worldwide to be putative wild reservoir hosts for different species of *Leishmania* (Roque and Jansen, 2014), however, to date, no studies demonstrated amastigote forms in tissues of bats. In fact, only molecular detection of *L. infantum* DNA have been reported in tissues of naturally infected bats, as seen in our study (Shapiro et al., 2013; Berzunza-Cruz et al., 2015; Castro et al., 2020).

In the present study, 20.4% of *A. planirostris* and 12% of *C. perspicillata* demonstrated positivity in the qPCR, without evidence of parasites by histopathological and immunohistochemical assays. The sensitivity obtained by Francino et al. (2006) was up to 0.001 parasites by qPCR reaction, which corresponds to less than 1 parasite/ml. However, constitutive parts of the parasites (fragments of DNA), nevertheless, despite demonstrating the presence of the parasite, it does not allow us to indicate its integrity (Silva et al., 2005; Roque and Jansen, 2014).

The absence of amastigotes forms and the nonspecific findings such discreet alopecia and the nonspecific focal inflammatory reaction in the wing skin of sampled bats could be associated with other etiologies (Bello-Gutiérrez et al., 2010; Martin et al., 2018). In fact, the histopathological analysis of wild South American mammals, such bush dog (*Speothos venaticus*) and opossum (*Didelphis albiventris*), naturally and experimentally infected respectively with *L. infantum,* revealed mild focal chronic dermatitis with the absence of amastigote forms (Sherlock et al., 1988; Figueiredo et al., 2008). It is noteworthy that the techniques that reveal the presence of leishmanias in skin fragments, as hemoculture, present low sensitivity but suggests transmissibility (Roque and Jansen, 2014).

The slight portal inflammation and vacuolation of hepatocytes observed in the sampled animals are unspecific changes and may not be associated with infection by *Leishmania* spp. (Molnár et al., 2008; Naidoo et al., 2016). Hepatic parenchyma has been reported to be apparently not a suitable site for initial *Leishmania* spp. expansion (Rodrigues et al., 2017). Additionally, Roque et al. (2010) did not find any inflammatory or degenerative changes in the small south American rodent *Thrichomys laurentius* experimentally infected by *L. infantum*. Furthermore, liver damage in bats that inhabit cities has been associated with intoxications by heavy metals and urban contaminants and pesticides (Ma, 1989; Bayat et al., 2014; Torquetti et al., 2021).

In our study, *L. infantum* positive bats showed minimal immune reaction in the splenic parenchyma, similarly observed in the negative animals. Roque et al. (2010) also reported minor changes without amastigotes in the splenic tissue of *T. laurentius* parasitized by *L. infantum*. Actually, intense tissue disorganization, disseminated inflammatory reaction and high parasite load have mainly been reported in spleen of dogs naturally infected by *L. infantum* (Santana et al., 2008; Cavalcanti et al., 2015; Bagues et al., 2018). The features found in the spleen of sampled bats indicated reaction to unspecific antigenic stimuli, because of exposure to a huge number of parasitic-infectious agents. As splenic parenchyma has a cellular diversity that allows the interaction among the immune system and a wide range of antigens (Santana et al., 2008), the routine histopathology may limit the study of parasitic-host interactions. In this sense, the use of IHC and situ hybridization techniques focused to detect subpopulations of inflammatory cells, as well as parasitic-infectious agents may contribute to a better understanding of the pathogenesis of leishmaniasis.

The results of autophagy and apoptosis, expressed by the presence of abundant TBMs, together with the absence of amastigote forms suggest that bats may control *L. infantum* infection. In fact, bats have been reported tolerant for several intracellular parasites (Sebghati et al., 2000; Hamilton et al., 2012; Schaer et al., 2013) since their immune systems recognize intracellular infections similarly to cellular injury caused during metabolic stress of flying and ageing (Brook and Dobson, 2015; Banerjee et al., 2020; Laing et al., 2019). Indeed, both flight physiology, ageing and infection by intracellular microparasites are known to cause the release of nitric oxide, a common form of reactive oxygen species, heavily implicated in the oxidative damage in cell membranes and consequent mechanisms of apoptosis and autophagy (Thomas and Suthers, 1972; Bogdan, 2001; Arnoult et al., 2009; Hanadhita et al., 2019). Our results, together with physiological characteristics of bats, suggest that, after infection, the newly parasitized cells undergo an autophagy process. Although we did not evaluate all tissues, our results allow us to hypothesize that phyllostomid bats seem to have an important role in reducing the risk of transmission, possibly acting as dead-end host. In order to understand the leishmania-bat relationship, future studies should investigate the presence of amastigotes and/or *Leishmania* DNA in bone marrow.

## Declaration of competing interest

None.
